# No-touch radiofrequency ablation using multiple electrodes: An in vivo comparison study of switching monopolar versus switching bipolar modes in porcine livers

**DOI:** 10.1371/journal.pone.0176350

**Published:** 2017-04-26

**Authors:** Won Chang, Jeong Min Lee, Jeong Hee Yoon, Dong Ho Lee, Sang Min Lee, Kyoung Bun Lee, Bo Ram Kim, Tae-Hyung Kim, Seunghyun Lee, Joon Koo Han

**Affiliations:** 1 Department of Radiology, Seoul National University Hospital, 101 Daehak-ro, Jongno-gu, Seoul, Korea; 2 Institute of Radiation Medicine, Seoul National University Medical Research Center, 103 Daehak-ro, Jongno-gu, Seoul, Korea; 3 Department of Pathology, Seoul National University Hospital, 101 Daehak-ro, Jongno-gu, Seoul, Korea; Texas A&M University, UNITED STATES

## Abstract

**Objective:**

To evaluate the in vivo technical feasibility, efficiency, and safety of switching bipolar (SB) and switching monopolar (SM) radiofrequency ablation (RFA) as a no-touch ablation technique in the porcine liver.

**Materials and methods:**

The animal care and use committee approved this animal study and 16 pigs were used in two independent experiments. In the first experiment, RFA was performed on 2-cm tumor mimickers in the liver using a no-touch technique in the SM mode (2 groups, SM1: 10 minutes, n = 10; SM2: 15 minutes, n = 10) and SB-mode (1 group, SB: 10 minutes, n = 10). The technical success with sufficient safety margins, creation of confluent necrosis, ablation size, and distance between the electrode and ablation zone margin (DEM), were compared between groups. In the second experiment, thermal injury to the adjacent anatomic organs was compared between SM-RFA (15 minutes, n = 13) and SB-RFA modes (10 minutes, n = 13).

**Results:**

The rates of the technical success and the creation of confluent necrosis were higher in the SB group than in the SM1 groups (100% vs. 60% and 90% vs. 40%, both *p* < 0.05). The ablation volume in the SM2 group was significantly larger than that in the SB group (59.2±18.7 cm^3^ vs. 39.8±9.7 cm^3,^ *p* < 0.05), and the DEM in the SM2 group was also larger than that in the SB group (1.39±0.21 cm vs. 1.07±0.10 cm, *p* < 0.05). In the second experiment, the incidence of thermal injury to the adjacent organs and tissues in the SB group (23.1%, 3/13) was significantly lower than that in the SM group (69.2%, 8/13) (*p* = 0.021).

**Conclusion:**

SB-RFA was more advantageous for a no-touch technique for liver tumors, showing the potential of a better safety profile than SM-RFA.

## Introduction

Radiofrequency ablation (RFA) is widely accepted as a nonsurgical option for treating primary and metastatic hepatic tumors, a potentially curative treatment for early-stage hepatocellular carcinoma (HCC), and as a bridge therapy for patients waiting for a liver transplant [1–5]. Furthermore, according to the recent Barcelona Clinic Liver Cancer Staging and Treatment Strategy guidelines for HCCs, RFA is favored over surgical resection for very early stage HCCs (single nodule <2 cm) [3]. However, one of the major disadvantages of RFA is a higher local tumor progression rate when compared to conventional surgery [6, 7]. Conventionally, RFA is performed by inserting a monopolar electrode into the tumor. However, it is not always possible to generate an ablation zone with a sufficient peritumoral margin (>5 mm) when using single monopolar electrode, and so it is often necessary to perform multiple ablations with overlapping techniques or ablations with multiple electrodes [8]. Various methods have been used to generate larger and more uniform ablation zones in a given time duration. These include switching ablation with or without a multipolar approach, modifying tissue characteristics using saline perfusion, and combination therapy with RFA or other therapies such as arterial chemoembolization [8–12]. However, despite their success in generating a sufficient ablation margin, these approaches also increased treatment complexity and complications [8]. The use of multiple ablation electrodes or multiple overlapping techniques can increase the risk of tumor seeding along the puncture route particularly for tumors located on the liver surface; however, this risk can be lowered by the use of tract ablation [13–16].

Recently, a number of studies [17–20] demonstrated the feasibility of multipolar RFA using multiple electrodes with no-touch techniques, and the promising outcomes of the no-touch RFA included high technical success, local tumor progression-free survival rates, and the absence of tract seeding episodes in HCC treatment. To avoid direct puncturing of the tumors, no-touch RFA is performed by inserting multiple electrodes outside of the tumor tissue. Thus, the risk of tract seeding is extremely low and a sufficient peritumoral margin is achievable. However, this technique requires a relatively long ablation time (18.5–27.4 minutes) and a significant amount of radiofrequency energy, which may result in parenchymal damage outside of the target tumor [17–20]. In monopolar RFA, the current spreads from each electrode centrifugally to the periphery and treatment was performed with a single electrode, whereas during bipolar RFA the electrical current flows between a pair of electrode. So theoretically, delivery of a high-density current into target tissues could be obtained more quickly in a bipolar mode than a monopolar mode, and subsequently, the ablation of tissues lateral to the electrodes could be reduced [8]. Although increased ablation efficiency is achieved using multipolar or switching bipolar RFA [21–23], a comparison between monopolar and bipolar RFAs for the no-touch technique has not been assessed in vivo.

Therefore, we evaluated the in vivo technical feasibility, efficiency, and safety of switching bipolar (SB) and switching monopolar (SM) radiofrequency ablation (RFA) as a no-touch ablation technique in the porcine liver.

## Materials and methods

This study received technical support and a research grant from STARmed Co. (Goyang, Republic of Korea). All authors had complete control of all the experimental data and information submitted for publication at all times. The funders had no role in study design, data collection and analysis, decision to publish, or preparation of the manuscript.

### The RFA equipment

**Fig 1:**
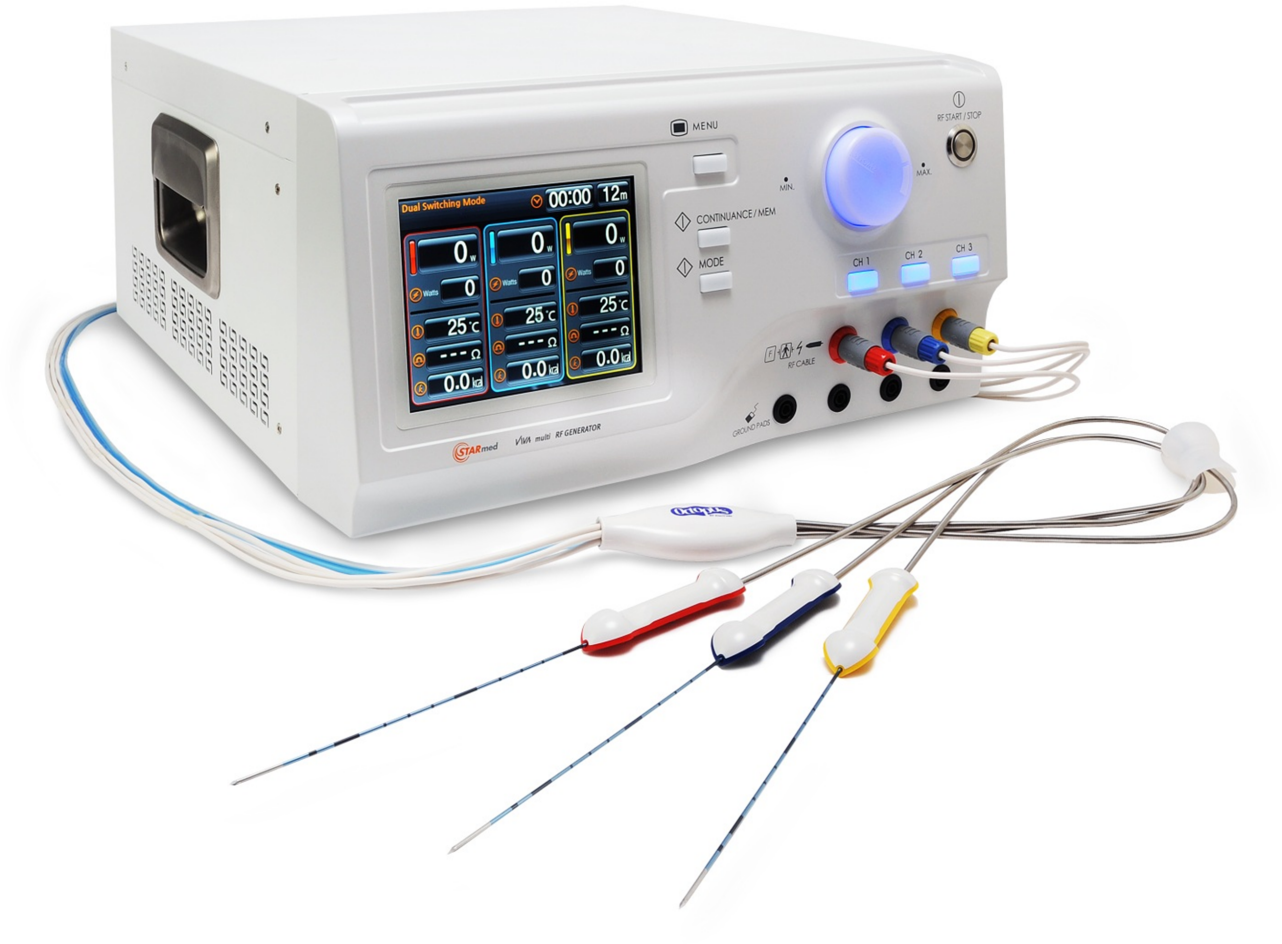
Photograph of a prototype RFA generator and a clustered separable Octopus® electrode.

A prototype of the multichannel radiofrequency (RF) system was developed to deliver RF energy in the SB mode using three electrodes. This allows the automatic switching of energy to one electrode or electrode pair depending on changes in electrical impedance, using the inherent “off time” of the power pulsing algorithm [8, 11, 24]. If the impedance of an active electrode or electrode pair increased to 50 ohms above baseline impedance, the energy delivery was switched to the inactive electrode pair or electrode [25]. A separable clustered electrode (Octopus® electrode; STARmed, Goyang, Korea) with three internally cooled electrodes and a 2.5-cm long active tip, was used for the no-touch technique [25] (Fig 1).

Chilled normal saline solution was infused into the lumen of each electrode and a peristaltic pump (VIVA Pump; STARmed, Goyang, Korea) was used to ensure the tip temperature remained below 25°C. Technical parameters such as average power output, electrical impedance, currents applied, and total energy delivered were monitored continuously and recorded using a monitoring software (VIVA Monitor Software V 1.0; STARmed, Goyang, Korea).

### Animals, anesthesia, and surgery

The study was approved by our Institutional Animal Care and Use Committee and all experiments were conducted in accordance with institutional guidelines. A total of 16 domestic male pigs (mean weight, 65 kg; range 60–70 kg) were used in our in vivo studies.

Each animal was sedated with an intramuscular injection of zolazepam (5 mg/kg, Zoletil; Virbac, Carroscedex, France) and xylazine (10 mg/ kg, Rompun; Bayer-Schering Pharma, Berlin, Germany), and the animals were intubated and ventilated during the procedures. Anesthesia was maintained by the inhalation of 1%-4% isofluorane (IsoFlo®; Abbott Laboratories, North Chicago, IL) in pure oxygen gas with mechanical ventilation. The pigs were placed in the supine position and a midline incision was made after sterile draping. One of the authors (W.C., with five years of experience in the RFA procedure and experiments) performed the ablation procedures through the midline incision under the guidance of ultrasonography (6–12 MHz linear transducer; Accuvix XQ; Medison, Seoul, Republic of Korea). Two to four ablation zones were generated in the liver of each animal; however, only one ablation was performed in each lobe of the liver. Therefore, a total of 56 ablation zones were created in 16 pigs. The animals’ vital signs, including pulse rate, electrocardiogram, and temperatures, were carefully monitored during the entire procedure.

### Tumor mimickers

**Fig 2:**
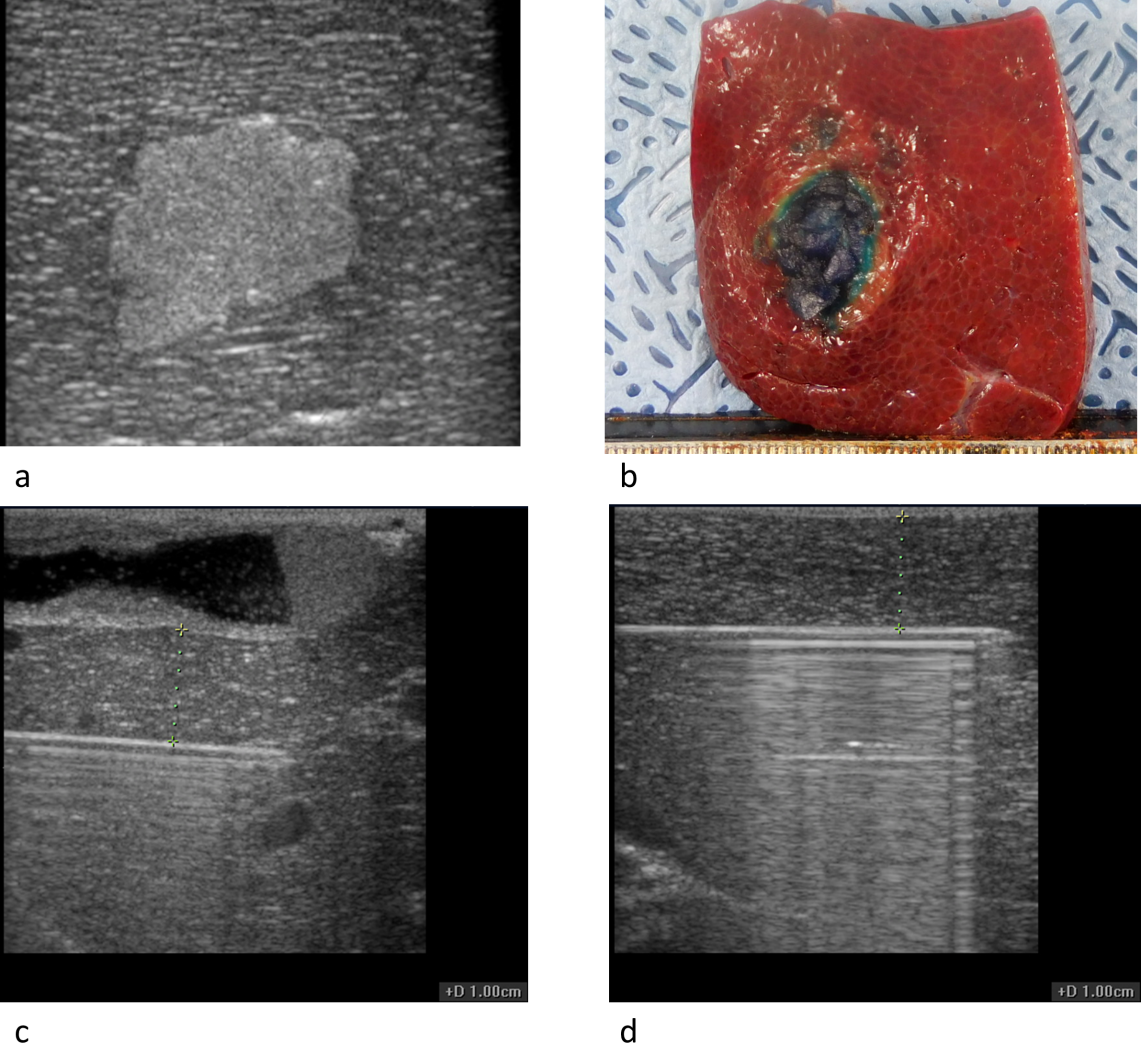
(a) Ultrasonograph of the agarose-based tumor-mimicker. (b) Photograph of the tumor mimicker on a sliced specimen. Ultrasonographs of an electrode inserted 1 cm apart from (c) the gallbladder and (d) liver surface. Electrodes were inserted parallel to the liver surface or gallbladder where possible.

For simulating the no-touch tumor ablation technique, tumor mimickers were made using a mixture of agarose, cellulose, glycerol and methylene blue as previously reported [26]. To create a 2 cm sized spherical or elliptical mass, approximately 2.5 cc of the mixture was injected using a 18G spinal needle into the porcine liver under the guidance of ultrasonography. In order to avoid the heat sink effect, care was taken not to inject tumor mimickers in the vicinity of large vessels. Tumor mimickers were detected *via* ultrasonography as hyperechoic lesions and blue nodules on gross specimens (Fig 2). Three perpendicular diameters of each tumor mimicker were measured using the electronic caliper on the ultrasonograph, and the volume was calculated by approximating the shape of the lesion to an ellipsoid.

### In vivo experimental setting

We performed two in vivo experimental studies to compare the efficiency and safety of performing no-touch RFA techniques in porcine livers, using SM and SB energy delivery modes. Experiment 1 compared the feasibility and efficiency of SM- and SB-RFA using no- touch ablation techniques by means of assessing technical success and ablation zone. Tumor mimickers were generated and injected as previously described. Three electrodes were inserted around the tumor mimicker through a triangular acryl plate containing multiple holes to maintain an interelectrode distance of 2.5 cm [25].

Experiment 2 was conducted to evaluate the safety of SM- and SB-RFA techniques. As a safety parameter, we checked the presence of thermal injury in adjacent organs or structures including the stomach, gallbladder, small bowel, and biliary tract at each segmental level. One of the electrodes was inserted in the liver 1 cm away from the liver surface, gallbladder or biliary tract (Fig 2). The other two electrodes were inserted through the same acryl plate used in experiment 1, thereby ensuring an inter-electrode interval of 2.5 cm. Temperature at the center of each ablation zone was measured in real-time using a thermometer placed at the center of the ablation zone. Moreover, times to reach tissue temperatures of 50°C, 60°C, 70°C, 80°C, and 90°C were recorded.

### Ablation protocols

In experiment 1, a total of 30 ablation lesions were made in either the SM mode (n = 20) or SB mode (n = 10). RF energy was applied in the SM mode for 10 minutes (group SM1, n = 10) or 15 minutes (group SM2, n = 10) and in the SB mode for 10 minutes (group SB, n = 10). The modes of the ablation were randomized after inserting electrodes to minimize a selection bias and other variations, including potential heat sink effects. The maximum RF delivered energy was 200 W in the SM mode and 100 W in the SB mode. The smaller energy level and 10-minute ablation time used in the SB mode were based on previous studies reporting that bipolar RFA could be performed with a relatively faster ablation time a better energy delivery efficiency than monopolar RFA [8, 21, 23]. In our clinical RFA practice we usually use 10~15 minutes ablation time as a single RF energy application unit with SM mode. Therefore, we evaluated the ablative efficiency of SM mode in two subgroups using the same 10 minutes ablation time, as well as a longer 15 minutes ablation time; these subgroups were SM1 and SM2, respectively [21, 23].

In experiment 2, thermal injury was compared between 15 minutes SM ablation and 10 minutes SB ablation modes. The maximum RF energy and ablation times were the same as in experiment 1. The rates of thermal injury in our previous *ex vivo* study were 30% and 100% in SB-RFA and SM-RFA using the same ablation time. However, we considered the heat sink effect might decrease the rates to 20% and 80% in SB-RFA and SM-RFA, respectively. The sample size was calculated with 0.05 of alpha and 0.20 of beta and therefore 13 ablations per group were required. We performed 26 ablations and the presence of thermal injury in the stomach (n = 4), gallbladder (n = 4), small bowel (n = 3) and segmental intrahepatic biliary tract (n = 2) were checked in each group.

### Assessment of technical success and ablation zone

All animals were euthanized by intravenous injection of potassium chloride immediately after the RFAs were performed.

**Fig 3:**
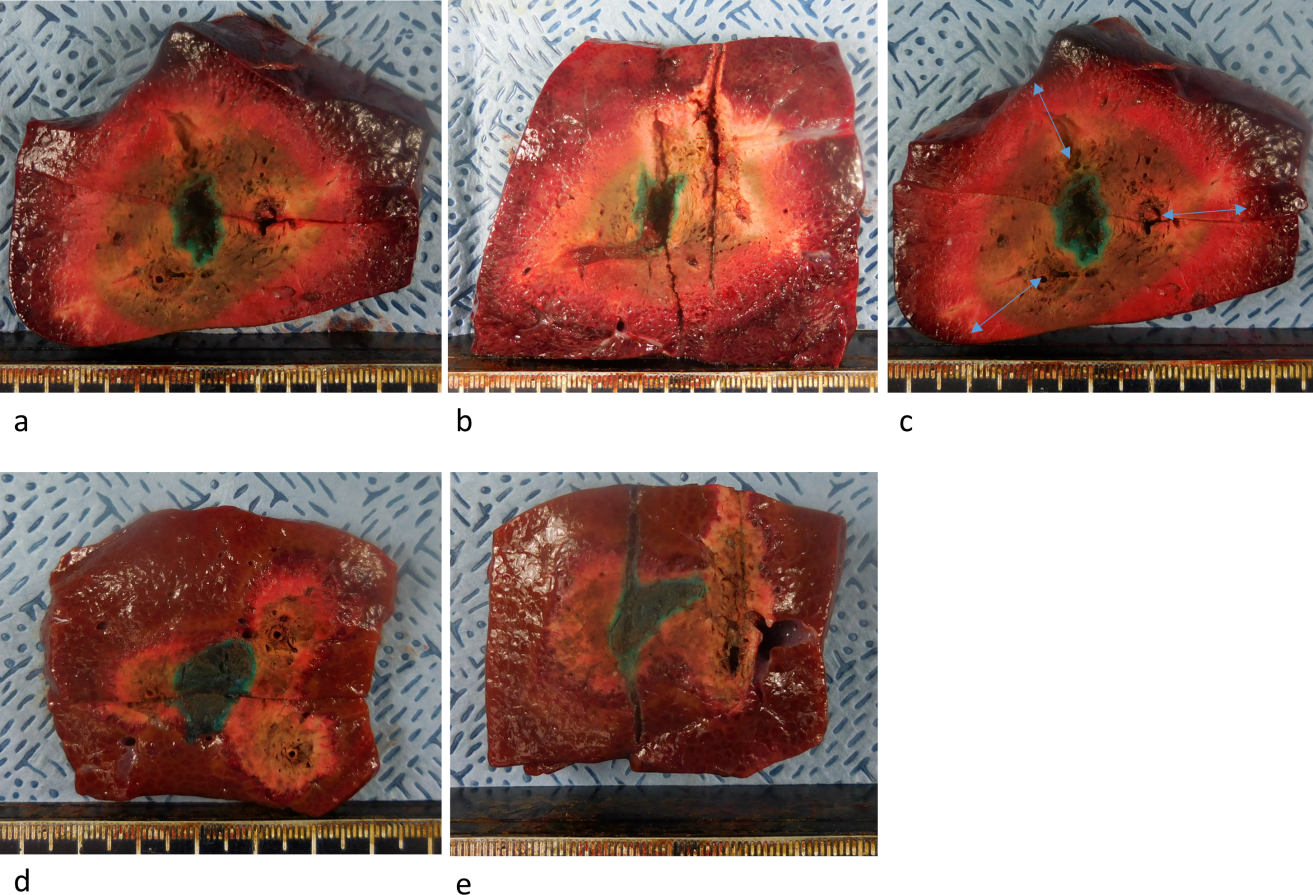
Photographs showing transverse and vertical planes of the specimen. (a, b) technical success with confluent necrosis (in the SB group). (d, e) technical failure with partial necrosis (in the SM2 group). (c) same specimen as shown in Fig 3a after TTC treatment. Arrows indicate the distance between the outer margin of the ablation zone and the electrode.

The livers were removed en bloc and hepatic segments containing ablation zones were excised along the electrode tract, then sliced in the transverse plane perpendicular to the electrode tract axis at 5–7 mm intervals so that sections included the largest areas of ablation and tumor mimicker. To prevent any bias in the measurements of ablation size, slices were photographed beside a ruler on a copy stand using a digital camera (Nikon Coolpix S6900; Nikon Inc., Tokyo, Japan). Two observers (W.C. and a technician, with 5 years and 10 years of experience in RFA experiments) measured the vertical diameter (Dv) in the vertical plane, the long-axis diameter (Dmx) and the short-axis diameter (Dmi) of RF-induced ablation zones at the transverse plane with the maximum area in consensus [27]. Technical success of the no-touch technique, size and shape of the ablation zone were evaluated. A technical success in terms of ablation was defined as >5-mm peritumoral ablation margins outside the tumor mimicker in all directions on the slices [28–30] (Fig 3).

Specimens were stained with 2% 2,3,5-triphenyl tetrazolium chloride (TTC) (Sigma-Aldrich, St Louis, MO) for 30 minutes at 20–25°C to assess cell viability. Distances between the electrode and outer margin of ablation zone (DEM) were measured on the plane with the maximum coagulation area (Fig 3). If the ablation zone reached the liver surface, DEM near the saturated surface was not measured, and the mean value of the measurable DEMs was calculated (Fig 3). In cases with confluent or partial confluent necrosis, the shape of the RF-induced ablation zone was quantitatively evaluated on the same plane using the ratio between the Dmi and Dmx and the circularity defined by the following formula: circularity = 4πA/P^2^, where A is the area of the measured zone and P is the perimeter of the area [23]. All measurements of diameter, distance, and circularity were performed on image files of the slices using Image J software (https://imagej.nih.gov). The volumes of ablation zones were only calculated when technical success was achieved. In the case of confluent ablation, the volume was calculated using the following formula by approximating the shape of the lesion to an ellipsoid: π (Dv × Dmx × Dmi)/6. In cases where the ablation zone was non-confluent, an ellipse intersected by the tangent points of the coagulation zone was drawn; the maximum (Dmx-eff) and minimum (Dmi-eff) diameters of the ellipse were measured. The volumes of the ablation zones were evaluated using the following formula according to the same approximation of the shape to an ellipsoid: Volume = π/6 × Dmx-eff × Dmi-eff × Dv. In order to compare the variability in ablation volumes among the three groups, the coefficient of variation of the ablation volume was calculated as the ratio of the standard deviation to the mean value of gross ablation volume. The effective ablation volume (Volume-eff) was also calculated using the formula: Volume-eff = π/6 × Dmin^3^, where Dmin is the shortest diameter measured.

### Assessment of thermal injury

Thermal injury of adjacent organs or biliary tract was checked as a safety parameter. After sacrificing the animals, the adjacent stomach, small bowel, gallbladder and liver segment containing targeted biliary tract were resected and fixed in 40 g/L formaldehyde solution. Specimens were cut into 3-mm thick slices, embedded in paraffin, and stained with hematoxylin and eosin for analysis by light microscopy. One of the authors (K.B.L. with ten years of clinical experience in the interpretation of liver and gastrointestinal pathology) reviewed the slides for the presence and depth of thermal injury. The depth of thermal injury was recorded as the deepest layer with thermal injury for the stomach, small bowel, and gallbladder [31–34]. Although an electrode was inserted 1cm away from the liver surface, gall bladder or biliary tract using USG guidance, distances were re-measured on the excised liver specimens using same manner, previously described for DEM measurement. And whether the ablation zone was reached to the liver surface adjacent to the target organs or biliary tract was recorded.

### Statistical analysis

For each experiment, the results were presented as the mean value ± standard deviation (SD). In experiment 1, results were compared between the SM-RFA groups (SM1 and SM2) and the SB-RFA group (SB) and then subgroup analyses among these three groups (SM1, SM2, and SB) were performed. The measured and calculated values as well as the monitored technical parameters were compared by t-test with unequal variances and the analysis of variance (ANOVA) test with Scheffe’s method as a post-hoc analysis. Regarding the rates of technical success and creation of confluent necrosis, we used the chi-square test and Marascuilo procedure for multiple comparisons of proportions [35]. The rate of thermal injury was compared using the chi-square test; and times to reach certain temperatures were compared using the t-test with unequal variances. For all statistical analyses, *p*-values < 0.05 were considered statistically significant. Statistical analyses were performed using the MedCalc statistical software, version 12.2.1 (MedCalc Software, Mariakerke, Belgium).

## Results

### Technical parameters and tumor mimickers

The maximum diameters of tumor mimickers were 1.99 ± 0.25 cm, 2.04 ± 0.15 cm, and 2.08 ± 0.20 cm, and their volumes were calculated to be 2.78 ± 0.68 cm^3^, 2.41 ± 0.69 cm^3^, and 2.42 ± 0.50 cm^3^ in the groups SM1, SM2, and SB, respectively. Despite efforts to avoid perivascular areas during implantation of tumor mimickers, we found that 30% (3/10), 40% (4/10), and 30% (3/10) of mimickers were contiguous to large vessels (> 3 mm) in the groups SM1, SM2, and SB, respectively [36].

The mean electrical impedance of SM mode was significantly lower than that of SB mode (all *p*-values < 0.001) (Table 1 and S1 Table). The average power and total amounts of energy delivered were significantly lower in the SB mode than in the SM mode (all *p*-values < 0.001).

**Table 1:** Measured values of technical parameters according to the power application modes.

Parameters	Group SM1 (n = 10)	Group SM2 (n = 10)	Group SB (n = 10)	SM1 vs. SM2	SM1 vs. SB	SM2 vs. SB
Total energy delivered (Kcal)	11.2 ± 0.7	16.2 ± 2.0	8.2 ± 0.8	< 0.001	< 0.001	< 0.001
Average power (W)	105.7 ± 5.8	102.2 ± 7.8	79.1 ± 4.4	0.266	< 0.001	< 0.001
Impedance (Ohm)	65.3 ± 8.2	63.3 ± 7.0	84.9 ± 12.3	0.563	< 0.001	< 0.001

Note–SM = switching monopolar, SB = switching bipolar.

There were no significant differences in the maximum diameter and volumes of tumor mimickers among the groups (*p* = 0.580 and 0.264, respectively).

### Technical success, size of ablation, and shape analysis

#### Technical success

A 100% (10/10) technical success was achieved using SB-RFA, which was significantly higher than the success of SM-RFA (SM1 and SM2, 65% [13/20], *p* = 0.0357). Moreover, subgroup analysis determined the technical success rate to be higher in the SB group than in the SM1 groups (100% [10/10] vs. 60% [6/10], p < 0.05). Perivascular tumor mimickers reduced the technical success rates to 0% (0/3), 25% (1/4), and 100% (3/3) in the groups SM1, SM2, and SB, respectively. Technical success rate for perivascular tumor mimickers was higher in the SB group than in the SM2 and SM1 groups (p<0.05). When cases of perivascular tumor mimickers were excluded, the technical success rates increased to 85.7% (6/7) and 100% (6/6) in the SM1 and SM2 groups and statistically not different among the three groups. The rate of confluent necrosis was higher in the SB group than in either the SM1 group (90% [9/10] and 40% [4/10], *p* < 0.05) or SM2 group; however, the difference in the rate between the SB and SM2 groups was not statistically significant (Table 2 and S1 Table, *p* > 0.05).

**Table 2:** Results of the technical success rate and shape analysis of RF-induced ablation zones in each group.

Parameters	SM-RFA (n = 20)	SB-RFA (n = 10)	*p-*value	Group SM1 (n = 10)	Group SM2 (n = 10)	Group SB (n = 10)	*p-*value		
							SM1 vs. SM2	SM1 vs. SB	SM2 vs. SB
Qualitative analysis of Coagulation Necrosis									
Technical Success	65% (13/20)	100% (10/10)	0.0357	60% (6/10)	70% (7/10)	100% (10/10)	NS	< 0.05	NS
Confluent necrosis	55% (11/20)	90% (9/10)	0.0595	40% (4/10)	70% (7/10)	90% (9/10)	NS	<0.05	NS
Partial confluent necrosis	35% (7/20)	10% (1/10)	0.1512	40% (4/10)	30% (3/10)	10% (1/10)	NS	NS	NS
Separated necrosis	10% (2/20)	0% (0/10)	0.3088	20% (2/10)	0% (0/10)	0% (0/10)	NS	NS	NS
Quantitative analysis of Coagulation Necrosis									
Circularity	0.86 ± 0.08	0.91 ± 0.03	0.027	0.85 ± 0.07	0.87 ± 0.09	0.91 ± 0.03	0.234		
Dmi/Dmx Ratio	0.88 ± 0.05	0.86 ± 0.10	0.982	0.84 ± 0.11	0.88 ± 0.09	0.86 ± 0.10	0.756		

Note–SM = switching monopolar, SB = switching bipolar, Dmx = maximum diameter of the ablative zone, Dmi = minimum diameter of the ablative zone, NS = not significant.

#### Measurements of ablation size

The volume of gross ablation was significantly smaller with SB- than with SM2-RF (39.8 ± 9.7 cm^3^ and 59.2 ± 18.7 cm^3^) (Table 3 and S1 Table). The overall DEM was lower in SB-RFA than in SM-RFA (1.31 ± 0.19 cm, and 1.07 ± 0.10 cm, respectively, *p* < 0.001). DEMs of SM1, SM2, and SB were 1.22 ± 0.14 cm, 1.39 ± 0.21 cm, and 1.07 ± 0.10 cm, respectively, and the SB group had a significantly lower DEM than the SM2 group (*p* < 0.05). Other size parameters including diameters and effective ablation volume were not significantly different between the groups.

**Table 3:** Results of ablation size measurement in each group.

Parameters	SM-RFA (n = 20)	SB-RFA (n = 10)	*p-*value	Group SM1 (n = 10)	Group SM2 (n = 10)	Group SB (n = 10)	*p-*value	SM1 vs. SM2	SM1 vs. SB	SM2 vs. SB
Dmx (cm)	4.91 ± 0.72	4.54 ± 0.33	0.114	4.57 ± 0.55	5.19 ± 0.76	4.54 ± 0.33	0.052			
Dmi (cm)	4.33 ± 0.91	3.91 ± 0.53	0.188	3.87 ± 0.87	4.71 ± 0.79	3.91 ± 0.53	0.209			
Dv (cm)	4.32 ± 0.65	4.27 ± 0.56	0.774	4.07 ± 0.63	4.53 ± 0.62	4.27 ± 0.56	0.383			
Gross Ablation volume (cm^3^)	50.0 ± 19.5	39.8 ± 9.7	0.117	39.3 ± 15.3	59.2 ± 18.7	39.8 ± 9.7	0.023	NS	NS	< 0.05
Effective Ablation volume (cm^3^)	34.3 ± 12.7	29.5 ± 10.5	0.331	27.1 ± 12.6	40.4 ± 9.7	29.5 ± 10.5	0.863			
DEM (cm)	1.31 ± 0.19	1.07 ± 0.10	< 0.001	1.22 ± 0.14	1.39 ± 0.21	1.07 ± 0.10	0.002	NS	NS	< 0.05
CV of the volume (%)	39	24.2		38.9	31.6	24.2				

Note–SM = switching monopolar, SB = switching bipolar, Dmx = maximum diameter of the ablative zone, Dmi = minimum diameter of the ablative zone, Dv = vertical diameter of the ablative zone, DEM = distance between electrode and ablation zone margin, CV, coefficient of variation.

#### Quantitative analysis of ablation shape

Circularity was significantly higher when SB-RFA was used compared to that when SM-RFA was used (0.91 ± 0.03 vs. 0.86 ± 0.08, *p* = 0.027); however, a subgroup analysis did not reveal any significant differences between the groups.

### Time to reach specified temperatures

The times to reach 50°C, 60°C 70°C, 80°C, and 90°C were 94.0 ± 54.1, 159.4 ± 87.1, 228.3 ± 143.5, 310.3 ± 193.1, 396.3 ± 212.3 seconds in SM-RFA and 62.8 ± 44.0, 109.0 ± 76.1, 114.5 ± 54.4, 171.4 ± 80.1, 221.8 ± 94.3 seconds in SB-RFA and significantly shorter in the SB-RFA group than in the SM-RFA group (*p* = 0.015, 0.030 and 0.048 for 70°C, 80, and 90°C; *p* = 0.091 for 50°C and *p* = 0.102 for 60°C).

### Safety assessment

Mean distances between an electrode and liver surface or targeted biliary tract were 1.16 ± 0.11 cm (range: 0.99 cm– 1.35 cm) and 1.11 ± 0.09 cm (range: 0.94 cm– 1.28 cm) in SM-RFA and SB-RFA, respectively (*p* = 0.218)whilst we had tried to insert an electrode keeping 1cm away from targeted organ or biliary tract. When the ablation area was reached to the targeted liver surface, and biliary tract, thermal injury of the adjacent organs or biliary tract was always found.

**Fig 4:**
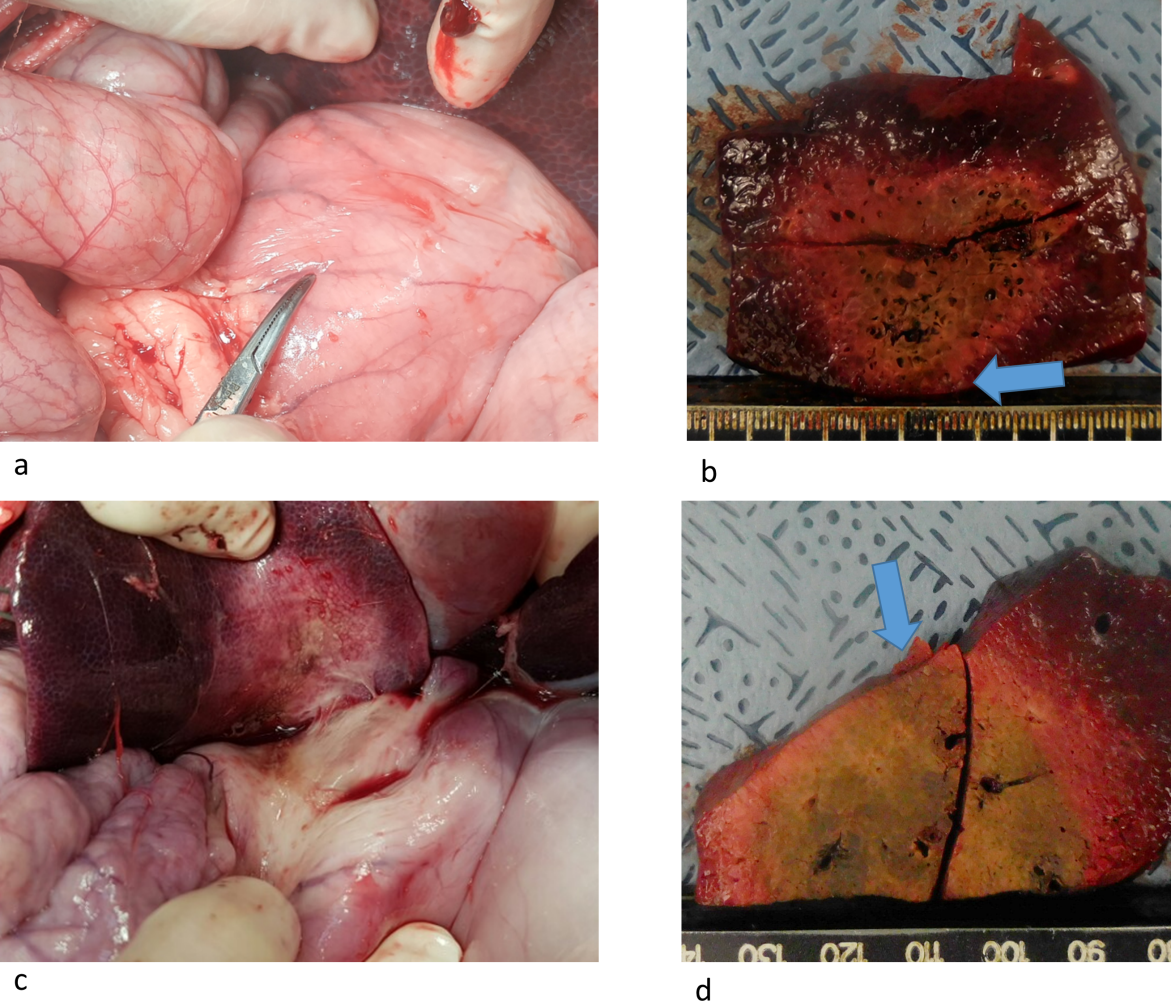
(a) Photograph showing the absence of stomach injury after SB-RFA. (b) Corresponding liver specimen, showing the ablation zone did not reach the liver surface abutting the stomach (arrow). (c) Photograph shows discolored thickened whitish area of the stomach suggesting thermal injury. (d) Corresponding liver specimen, showing the ablation zone reached the liver surface abutting the stomach (arrow).

**Fig 5:**
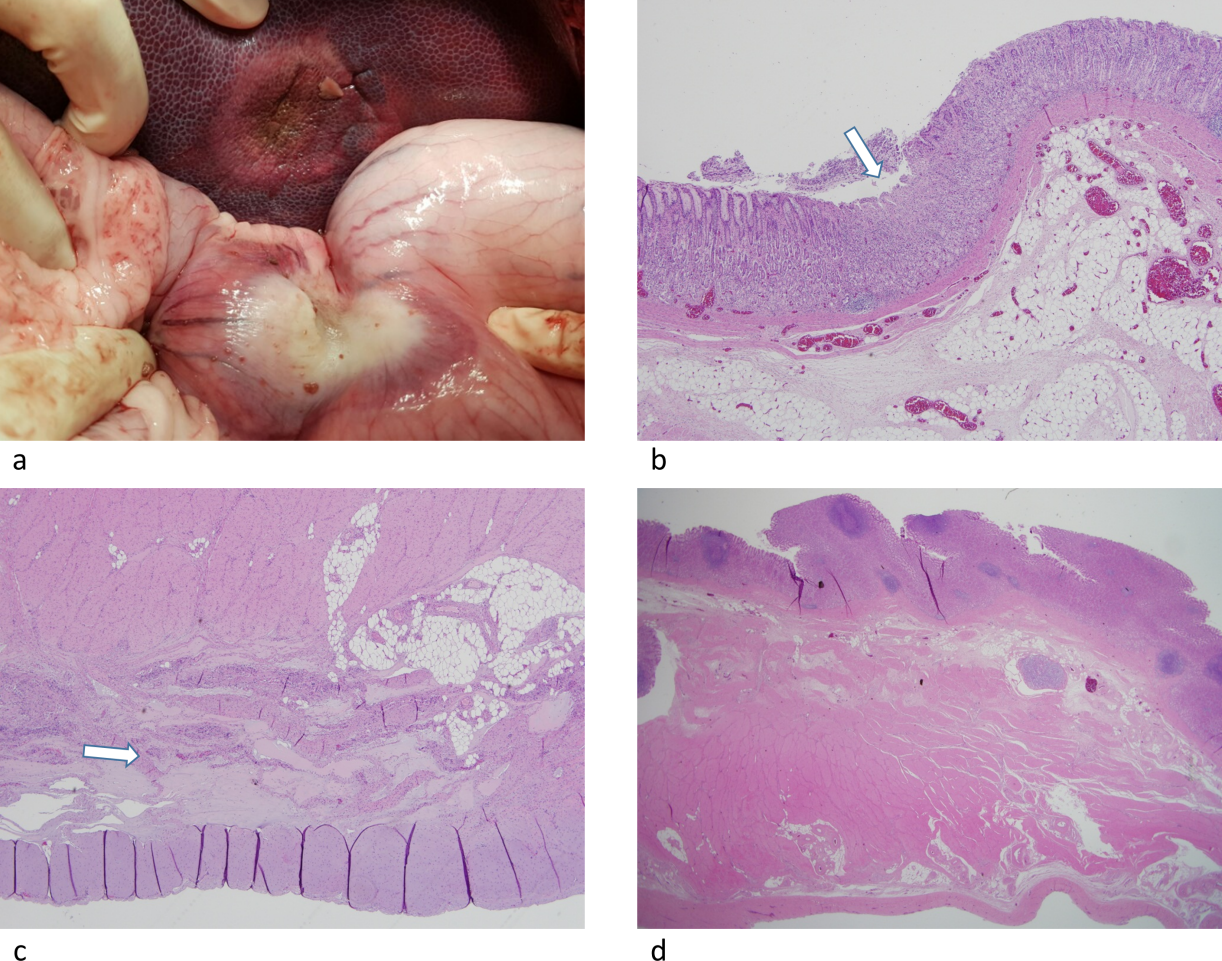
(a) Photograph shows discolored thickened whitish area of the stomach suggesting thermal injury. (b) (c) Corresponding stomach specimen with hematoxylin and eosin staining (H&E) shows thermal injury to the mucosa. Focal mucosal necrosis is present in (b) (arrow) (x40), and myxoid degeneration of serosa, subserosa and proper muscle with dilated lymphatic channels, and fluid accumulation in subserosa are present in (c) (arrow) (x40). (d) Photograph shows the H&E stained stomach specimen without thermal injury (x12.5).

**Fig 6:**
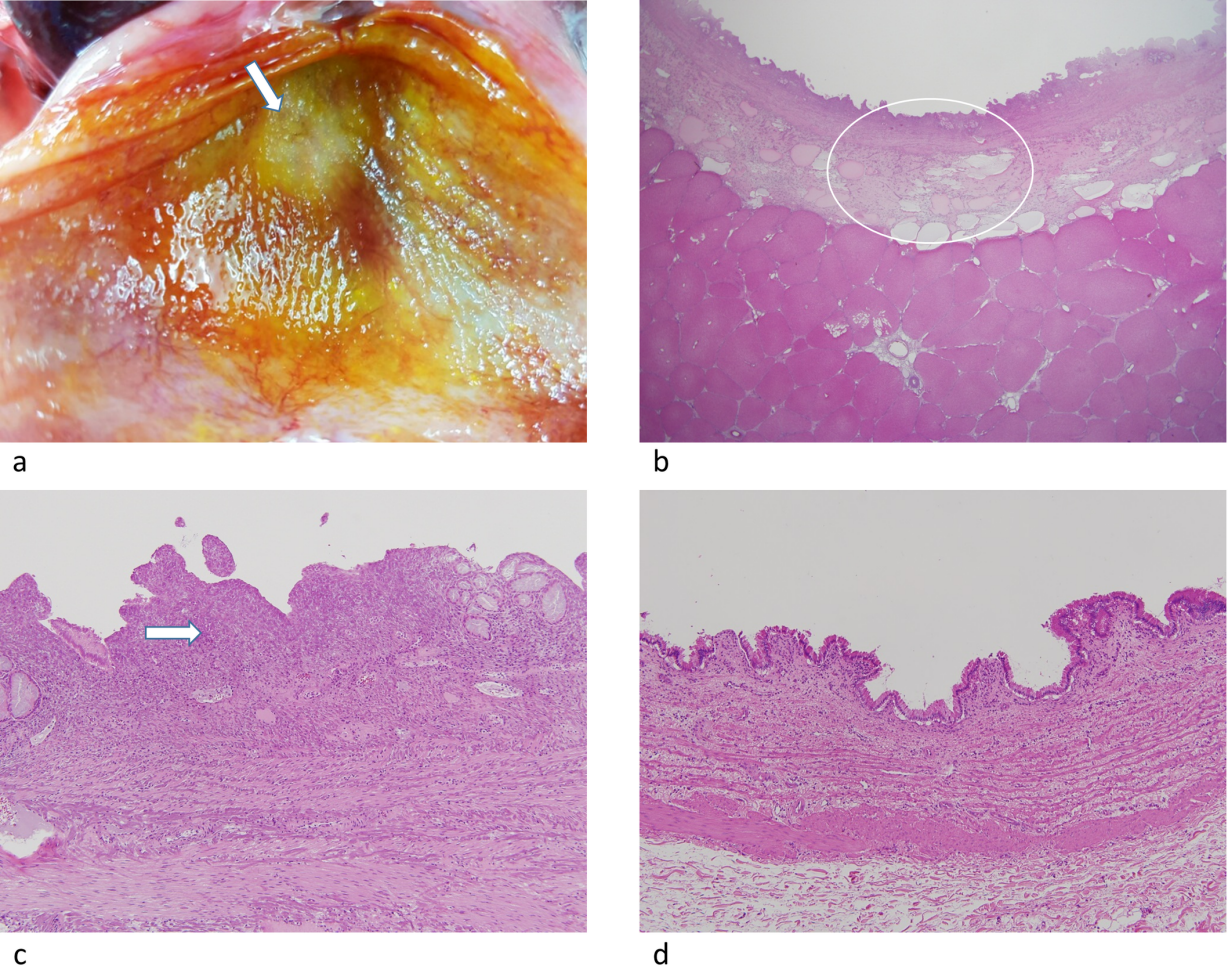
(a) Photograph shows the whitish area of the gall bladder suggesting thermal injury (arrow). (b) (c) Corresponding gall bladder specimen and adjacent liver parenchyma with hematoxylin and eosin staining (H&E) show thermal injury to the mucosa. Mucosal and submucosal damage are evident. Lymphatic dilatation of subserosa is noted in (b) (circle) (x12.5). The mucosal epithelium is replaced by dense fibrosis in (c) (arrow) (x100). (d) Photograph shows the H&E stained gall bladder specimen without thermal injury (x100).

**Fig 7:**
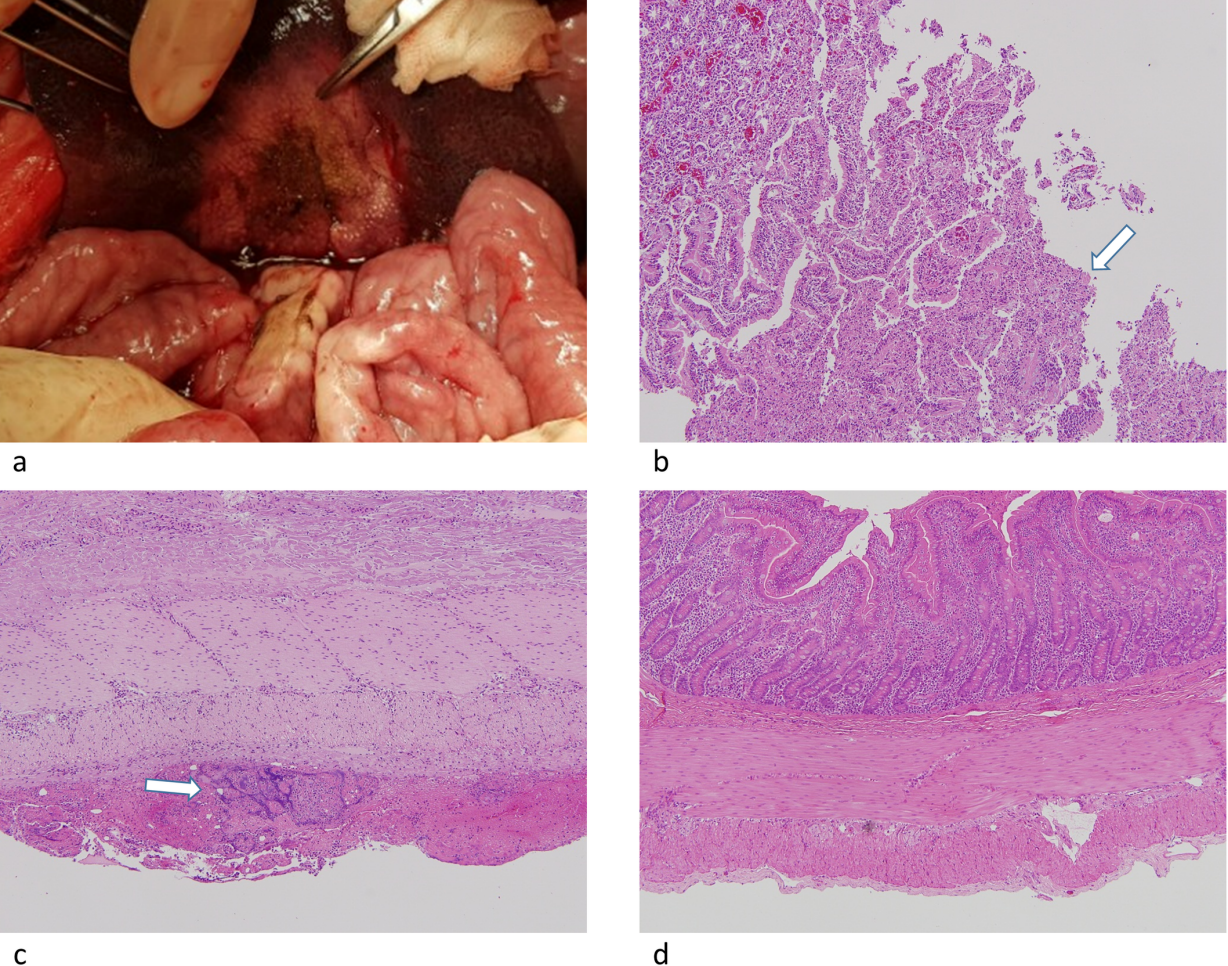
(a) Photograph shows discolored thickened whitish area of the small bowel suggesting thermal injury. (b) (c) Corresponding small bowel specimen with hematoxylin and eosin staining (H&E) shows thermal injury to the mucosa. Mucosal ulceration was noted in (b) (arrow) (x100). Chronic active inflammation in serosa and subserosa with focal necrosis are present in (c) (arrow) (x100). (d) Photograph shows the H&E stained small bowel specimen without thermal injury (x100).

Thermal injury to adjacent organs and biliary tracts was less frequently noted with SB-RFA than with SM-RFA (23.1% (3/13) and 69.2% (9/13), respectively, *p* = 0.021) (Table 4 and S2 Table; Fig 4). Excluding injury to the biliary tract, the rate of thermal injury penetrating the muscle layer was lower when SB-RFA was used compared to that when SM-RFA was used; however, these differences were not statistically significant (18.2% (2/11) and 54.5% (6/11), respectively, *p* = 0.084) (Figs 5–7).

**Table 4:** Thermal injury to the adjacent organs and structures in Each RFA mode.

Mode	Target	Thermal injury	Depth
SM-RFA	Stomach	75% (3/4)	Proper muscle (n = 1); Mucosa (n = 2)
	Gallbladder	50% (2/4)	Mucosa (n = 2)
	Small bowel	100% (3/3)	Proper muscle (n = 1); Submucosa (n = 1); Mucosa (n = 1)
	Biliary tract	50% (1/2)	
	Total	69.2% (9/13)	
SB-RFA	Stomach	25% (1/4)	Subserosa (n = 1)
	Gallbladder	50% (2/4)	Mucosa (n = 2)
	Small bowel	0% (0/3)	
	Biliary tract	0% (0/2)	
	Total	23.1% (3/13)	

Note–SM = switching monopolar, SB = switching bipolar.

## Discussion

Our in vivo study demonstrated that the no-touch RFA technique, using an Octopus electrode, was feasible for treating tumors using SM and SB energy delivery modes. With the exception of cases with perivascular tumor mimickers, technical success rates were 100% in both the SM2 and SB groups. However, the overall rates of technical success and confluent necrosis were significantly better in the SB group compared with the SM1 group. Technical success rates in SM-RFA could be increased with a longer ablation time. Technical success rate for the perivascular mimickers was increased in the SM2 group (25% (1/4)) compared to the SM1 group (0%, 0/3), but not statistically significant and still lower than that of the SB group (100% (3/3), p< 0.05). Therefore, in order to achieve confluent necrosis with SM-RFA, it might be necessary to perform additional ablations while changing the position of electrodes; however, this will increase the complexity of the procedure and related complications. Therefore, in single ablation session, a higher technical success rate using the no-touch technique may be achieved with SB-RFA rather than with SM-RFA.

The gross ablation volume was significantly larger in the SM2 group than in the SB group; DEM was significantly smaller in the SB group than in the SM2 group (p < 0.05). Although the previous ex vivo study showed DEM and gross ablation volume were significantly smaller in the SB group compared to the SM1 and SM2 groups [37], but in our result of this in vivo study, differences between SM1 and SB groups were not significant. Technical failure was determined as insufficient peritumoral margin with partial or separated necrosis and may be caused by the heat sink effect of adjacent vessels. Differences between ex vivo and in vivo studies showed heat sink effect in RFA. With respect to energy delivery, monopolar RFA was vulnerable to the heat sink phenomenon, and the presence of large peritumoral vessels increased the risk of incomplete ablation [36, 38], whereas, SB-RFA was less affected by the heat sink phenomenon compared with SM-RFA in the perfused ex vivo bovine liver model [39]. Since large-scale ablations around the perimeter of the tumor may be associated with a greater risk of thermal injury to adjacent structures, it is necessary to create a sufficiently large zone of confluent ablation zone to generate an adequate ablation margin; however it should not be too large as that could create unnecessary injury to the adjacent normal tissue [23]. In single ablation session, a higher technical success rate using the no-touch technique may be achieved with SB-RFA rather than with SM-RFA.

Theoretically, no-touch RFA techniques can provide several advantages over conventional tumor puncture RFA techniques, such as the absence of tract seeding or peritoneal seeding, and the absence of an increase in intratumoral pressure. However, the peritumoral ablation size could be larger for the no-touch RFA technique using multiple electrodes placed outside the tumor than for conventional RFA methods that puncture the tumor, and may therefore, increase the risk of thermal injury to adjacent structures. In the SM1 and SB groups, ablation time was the same, and ablation volume and DEM were not significantly different; however, the rates of technical success and confluent necrosis were significantly lower in the SM-RFA than in the SB-RFA group. According to previous studies, bipolar RFA has greater energy efficiency and enables a faster ablation time than monopolar RFA. The lower technical success observed in the SM-RFA group SM1 was in good agreement with these studies [21, 23]. Thermal injury was evaluated in the cases of successful ablation and ablations were compared between SM-RFA (15 minutes) and SB-RFA (10 minutes). No-touch SB-RFA created less frequent thermal injury to adjacent organs and structures than SM-RFA, although the differences in mean DEM between SM2 and SB groups was only 0.33 cm. Moreover, in cases where there were successful ablations in both SM- and SB-RFA groups, the ablation volume following SB-RFA was significantly smaller than that in the SM2 group, and it showed less ablation of the liver tissue outside of the electrode (shorter DEM). Conversely, the temperature at the center of the ablation zone had risen more quickly in the SB mode than in the SM mode. These results could be explained by the basic physical differences in electrical current flow between the SM and SB RF energy delivery techniques. In monopolar RFA, the current spreads from each electrode centrifugally to the periphery, whereas during bipolar RFA the electrical current flows between a pair of electrodes and prevents the ablation zone from perfusion-mediated tissue cooling effect, thereby resulting in a faster and more focal heating between the electrodes [8, 23]. In our study, although the delivered energy and power was significantly lower in SB-RFA group, ablation volume was not significantly different between the SM1 and SB groups, which suggest the higher energy efficiency of the SB-RFA[23]. And there is an increasing risk of skin burn related with ground pads, when large amount of RF current was used with multiple electrodes during the RFA procedure. Also, there have been theoretical concerns on RF energy induced damage in patients with metallic implants or cardiac pacemakers in monopolar RF ablation. But there has been no definite evidence supporting that dispersed energy through the patients used in RFA is related to the internal damage of the body near the metallic implants [40, 41]. Based on our results, we believe that the SB mode may be the optimal energy delivery mode for the no-touch RFA technique than the SM mode, because it effectively increases tissue temperature at the center, and induces lesser thermal injury to the surrounding organs.

RFA is widely accepted as one of the curative treatment options for early stage HCC in patients who are not good surgical candidates, primarily because it is more cost-effective and less invasive than surgery [2, 42]. Monopolar RFA is the most commonly used technique, and requires placement of the electrode within the target tumor. It creates an ablation zone centrifugally along the flow of electrical current in the tissue surrounding the electrode [27]. Risk of tract or peritoneal seeding after RFA is inevitable [15]; however, an increased risk of tumor seeding is associated with pericapsular tumors, poorly differentiated tumors, and high α-fetoprotein levels [43]. After Llovet et al. reported a high rate (12.5%, 4/32 patients) of tumor tract seeding when treating HCC with RFA [43], it has been raised as a major issue, particularly in patients treated for a curative purpose [44]. According to a systematic review of tumor seeding after percutaneous diagnostic and therapeutic procedures for HCC [14], the mean risk for tumor seeding after RFA alone was 1.73% (range, 0–5.56%), and after RFA with biopsy, the mean risk increased to 2.5%. Despite the relatively low risk of tumor seeding, and considering the cumulative risk of tract seeding for multisession RFAs or RFAs with multiple electrode insertions, the no-touch technique could be valuable for preventing tract seeding after RFA, particularly in patients waiting for liver transplants or patients with HCCs located on the liver surface [44, 45]. In order to avoid capsular breach during ablation, the no-touch wedge ablation technique may reduce the potential risk of tumor rupture and consequent hemorrhage [46]. Additionally, as the drainage vessels of HCC change from hepatic veins to peritumoral sinusoid or portal veins [47, 48], the no-touch technique can induce thrombosis in the draining peritumoral vessels which may be advantageous in decreasing the risk of metastases via the vessels. Until now, few studies have reported on the use of no-touch tumor ablation techniques using multipolar RFA with multiple bipolar electrodes, or multiple microwave antennae for liver tumors, especially for subcapsular tumors [17, 20, 45, 46]. When a traditional ablation technique using the monopolar mode with placement of electrode in the tumor has been used, the areas near the ablation probe are heated to greater than tumor-lethal temperatures; however, this is not as readily achieved at the periphery of the ablation zone. Therefore, residual tumor is often seen as scattered, nodular, or as eccentric enhancement at the margin of the ablation zone [49].

The present study has several limitations. First, the feasibility and safety of the no-touch technique were evaluated intraoperatively; therefore, we were unable to assess the feasibility and safety of percutaneous RFA. Thus, the feasibility of the no-touch technique in percutaneous RFA should be evaluated. Since the use of RFAs with multiple electrodes is routine in clinical practice, the differences in safety profile between the intraoperative and percutaneous approaches are expected to be minimal. Second, we did not compare our SB-RFA system with the multipolar RFA system, which shows promising results for no-touch ablation [19, 20]. Third, this in vivo study was performed in a relatively small number of animals, so we could not demonstrate significant differences in technical success between the SM2 and SB groups and differences in thermal injury in each adjacent organ which have different characteristics including heat sink effects. In the SM2 group, technical failure resulted from the perivascular location of tumor mimickers. Further studies to evaluate the adjacent organ injury and heat sink effect especially for perivascular tumors are required. Fourth, although SM-RFA with would be feasible with no-touch technique for the non-perivascular tumor, we could not suggest the optimal ablation time for SM-RFA. Because technical success rate for non-perivascular mimicker was 100% in SM RFA for 15 minutes, the optimal ablation time to cover the whole tumor mimicker and to reduce adjacent organ injury might be shorter than 15 minutes. Fifth, we only tested the no-touch technique with a single inter-electrode interval of 2.5 cm. Additional studies using larger interelectrode intervals would be valuable. Finally, the RF ablations were performed using a tumor mimicker; although there was no significant difference in tissue impedance and ablation size between groups with or without tumor mimickers [26], the thermal efficiency of the current RF system might not be translated into clinical practice owing to different tissue textures of target tumors.

In conclusion, our results demonstrate that SB-RFA with no-touch technique enables faster ablation with sufficient peritumoral margin and has potential to provide better safety profile with smaller adjacent parenchymal ablation zones, sufficient peritumoral margins, and lesser thermal injury to the adjacent organs compared with the SM-RFA techniques.

## Supporting information

S1 Table Raw data of technical parameters, technical success, size of ablalation and shape analysis. (XLSX)

S2 Table Raw data of adjacent organ injuries. (XLSX)
